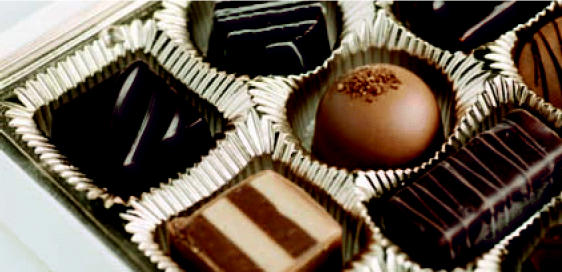# The Beat

**Published:** 2006-10

**Authors:** Erin E. Dooley

## Don’t Hold Your Breath for New Inhalers

In response to a federal push to remove chlorofluorocarbons (CFCs) from asthma inhalers by December 2008, some manufacturers have already stopped production of CFC-containing inhalers and are employing a new, more environmentally friendly propellant, hydrofluoroalkane. But the new inhalers are still in short supply, and drug stores are beginning to see a shortage. Why? The new inhalers can cost up to three times more than their predecessors, and some manufacturers are reluctant to pursue the switch wholeheartedly as long as the cheaper inhalers are available. For now, some experts suggest that asthma patients rely more on preventive treatments such as corticosteroids.

**Figure:**
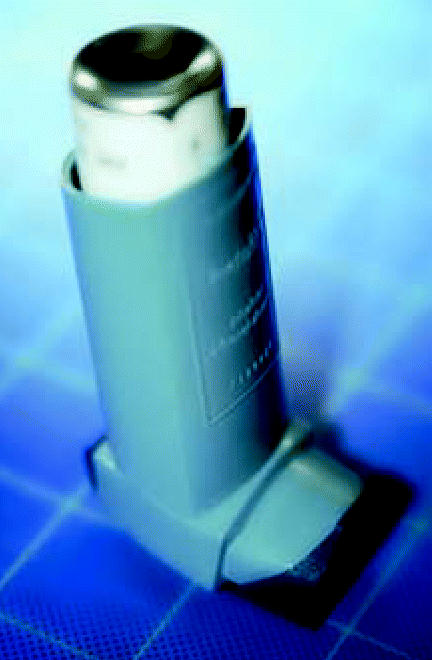


## A Yen for Better Farm Chemical Rules

Strict new rules for farm chemicals such as pesticides and veterinary medicines took effect in Japan on 1 June 2006. The rules establish maximum residue limits for 799 chemicals, more than triple the number previously regulated. A blanket limit of 0.01 ppm has been set for all other chemicals. The rules apply to all farm chemicals and all categories of food. Food producers and distributors, now striving to follow the regulations, are looking for ways to minimize pesticide drift from one crop to another and are setting up plans for more stringent inspection of products—even those that originate in other countries—before they hit store shelves.

## Sounding Off on School Noise

Many schools use sound amplification systems to help teachers be better heard in their classrooms, but according to a policy statement issued in June 2006 by the Acoustical Society of America, such systems actually make the situation worse. Use of sound systems increases overall sound levels and may be too loud for comfortable listening. Furthermore, in many situations, amplified sound can contribute to noise in neighboring classrooms. The society says the best way to improve noisy conditions is through renovation and better design of classrooms in compliance with American National Standards Institute S12.60-2002 standards for noise control in classrooms.

**Figure:**
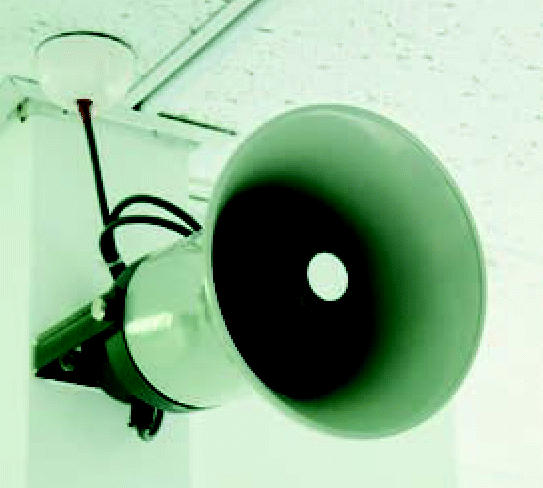


## Blue Planet Run

In the summer of 2007 Dow Chemical and the Blue Planet Run Foundation will cosponsor the first Blue Planet Run, a worldwide relay with runners coursing across 22 countries, 24 hours a day, for more than 3 months. The aim of the run is to raise awareness of and funds for sustainable projects to provide safe drinking water for people around the world. Waterborne diseases are the single largest contributor to human sickness and health-related deaths, with more than 25,000 people falling victim to unsafe water each day.

The foundation has already funded a number of projects—40 were implemented in 8 countries in 2005. The runs are planned to be a biannual event.

**Figure:**
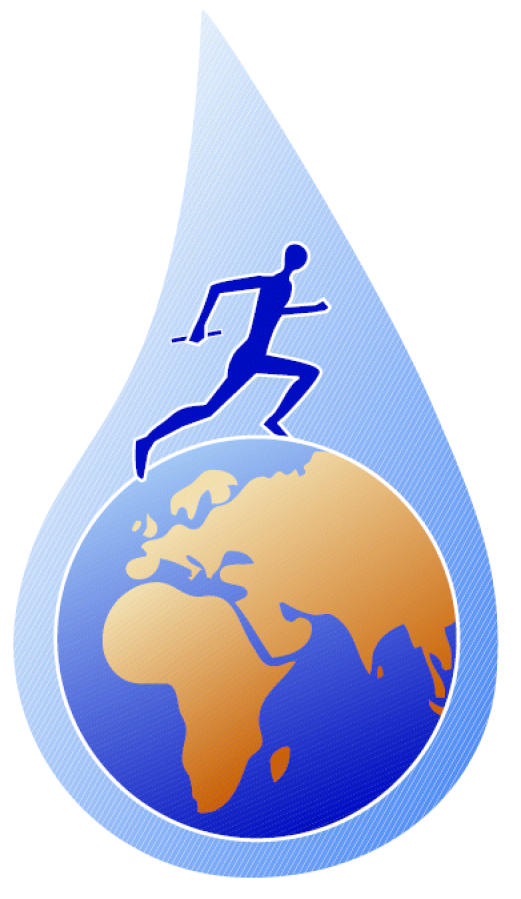


## Good Science for Girls

Nearly 90% of U.S. engineers are male. To help bridge this gender gap, the New Jersey Institute of Technology has developed Femme, a program of five-week summer classes to encourage girls in grades 4–9 to aspire to careers in science and engineering. For the summer of 2006 the classes focused on environmental, aeronautical, mechanical, chemical, and biomedical engineering. A related program for 9th graders, Femme Academy, consisted of intensive instruction in electrical and computer engineering. Conducted since 1981, Femme has seen nearly 70% of its alumnae study technology, science, or engineering at the college level.

## A Bevy of Biodegradables

In an effort to help stem the massive stream of waste dumped into landfills, new corn-based plastic bottles have recently hit the market in Great Britain and the United States, where millions of plastic bottles are used each day. The containers remain stable on store shelves, but decompose under composting conditions; they can also be recycled. British supermarket giant Sainsbury’s has also introduced biodegradable wrap and trays for organic foods, and other UK grocery chains are starting to use biodegradable films and grocery bags. Coca-Cola is looking into compostable bottles for its drinks as well, and Nestlé has come up with trays for chocolates sold in Britain that disintegrate on contact with water.

**Figure:**